# Characterization of drug resistance and genetic diversity of *Plasmodium falciparum* parasites from Tripura, Northeast India

**DOI:** 10.1038/s41598-019-50152-w

**Published:** 2019-09-23

**Authors:** S. J. Patgiri, K. Sarma, N. Sarmah, N. Bhattacharyya, D. K. Sarma, T. Nirmolia, D. R. Bhattacharyya, P. K. Mohapatra, D. Bansal, P. K. Bharti, R. Sehgal, J. Mahanta, A. A. Sultan

**Affiliations:** 1 ICMR - Regional Medical Research Centre, North East Region, Dibrugarh, Assam India; 2 Present Address: ICMR - National Institute for Research in Environmental Health, Bhopal, Madhya Pradesh India; 3 Department of Microbiology and Immunology, Weill Cornell Medicine-Qatar, Cornell University, Doha, Qatar; 4 ICMR - National Institute for Research in Tribal Health, Jabalpur, Madhya Pradesh India; 5 Department of Medical Parasitology, Postgraduate Institute of Medical Education and Research, Chandigarh, India; 6 Present Address: Ministry of Public Health, Doha, Qatar; 7 Present Address: Model Rural Health Research Unit (DHR), Assam, India

**Keywords:** Parasite genetics, Malaria

## Abstract

Monitoring of anti-malarial drug resistance is vital in Northeast India as this region shares its international border with Southeast Asia. Genetic diversity of *Plasmodium* parasites regulates transmission dynamics, disease severity and vaccine efficacy. *P*. *falciparum* chloroquine resistance transporter (*Pfcrt*), multidrug resistance-1 (*Pfmdr-1*) and kelch 13 propeller (*PfK-13*) genes which govern antimalarial drug resistance and three genetic diversity markers, merozoite surface protein 1 and 2 (*Pfmsp-1*, *Pfmsp-2*) and glutamate rich protein (*Pfglurp*) were evaluated from Tripura, Northeast India using molecular tools. In the *Pfcrt* gene, 87% isolates showed triple mutations at codons M74I, N75E and K76T. 12.5% isolates in *Pfmdr-1* gene showed mutation at N86Y. No polymorphism in *PfK-13 propeller* was found. Polyclonal infections were observed in 53.85% isolates and more commonly in adults (p = 0.0494). In the *Pfmsp-1* locus, the K1 allelic family was predominant (71.2%) followed by the 3D7/IC family (69.2%) in the *Pfmsp-2* locus. RII region of *Pfglurp* exhibited nine alleles with expected heterozygosity of 0.85. The multiplicity of infection for *Pfmsp-1*, *Pfmsp-2* and *Pfglurp* were 1.56, 1.31 and 1.06 respectively. Overall, the study demonstrated a high level of chloroquine resistance and extensive parasite diversity in the region, necessitating regular surveillance in this population group.

## Introduction

Northeast (NE) India, with a population of 45,772,188 (2011 census of India) contributes to approximately 28,341 malaria cases in a year, mostly due to *Plasmodium falciparum*^1^. In 2018, almost half (46.15%) of these cases were reported from the state of Tripura^1^. The NE region is strategically situated and shares a long international border with Southeast Asian countries such as Bhutan, Nepal, China, Myanmar and Bangladesh. This region is highly vulnerable for the importation of anti-malarial drug resistant strains of *P*. *falciparum* from surrounding Southeast Asian countries and thereby provides a gateway to the rest of India. The state of Tripura is highly endemic to malaria and a large outbreak was reported in 2014 with around 10,000 malaria cases and more than a hundred deaths^2^.

Many areas of NE region have stable malaria with a high asymptomatic carrier rate and high levels of transmission which increases the vulnerability of people to polyclonal infections^3^. Population movement to other parts of India where unstable malaria is prevalent disseminates new parasite strains resulting in malaria outbreaks and spread of drug resistance. Keeping in mind the diversity of the region and its proximity to malaria hot spots of Southeast Asia, drug resistance monitoring is vital. Chloroquine (CQ) was initially the most commonly used drug for presumptive treatment of all fever cases followed by 8-aminoquinoline for radical cure of falciparum malaria and to prevent relapses in vivax malaria^4^. CQ resistance has been linked to two parasite molecular markers: *P*. *falciparum* CQ resistance transporter (*Pfcrt*) gene located on chromosome 7 and *P*. *falciparum* multidrug resistance-1 (*Pfmdr-1*) gene situated on chromosome 5^5,6^. The Kelch 13-propeller (*K-13*) domain has been recently linked with artemisinin drug resistance both *in vitro* and *in vivo* and has been utilised widely since then for molecular surveillance^7^.

The information on genetic diversity of *Plasmodium* parasites is essential in developing an effective malaria vaccine because antigenic variation in these parasites significantly hinders vaccine research due to multiple alleles effectively evading vaccine-induced allele specific immunity. The merozoite surface protein 1 and 2 (*msp1* and *msp2*) and glutamate rich protein (*glurp*) of *P*. *falciparum* are potential candidate antigens for vaccine development^8,9^. Multiplicity of infection (MOI) is a parameter that is not only related to the transmission intensity of malaria but also predicts disease severity^10^. Infection by more than one parasite genotype has the potential to naturally select more virulent strains and thereby cause more severe infections^10^.

The current study examined the genetic diversity of *P*. *falciparum* parasites and distribution of anti-malarial drug resistance genes in Tripura state, NE India. The findings will help in understanding the current status of drug resistance genes and genetic structure of *P*. *falciparum* parasites which will improve existing and future malaria control strategies in the region.

## Results

### Study population

A total of 242 patients with fever (133 males, 109 females) were screened for malaria using microscopy and rapid diagnostic tests (RDT). Of these, 84 (males 53, females 31, *p* = 0.078, OR = 1.67) were found positive for malaria (*P*. *falciparum* infection 84.52%, *P*. *vivax* 10.71% and mixed infection 4.76%). Children aged ≤15 years had a higher rate of infection as compared to adults (36.11% vs. 28.57%, OR = 1.413), however the difference was not significant statistically (*p* = 0.2657). All cases positive by RDT and microscopy were also positive by nested Polymerase chain reaction (PCR) for species identification.

### Mutation analysis of drug resistance genes

*P*. *falciparum Pfcrt*, *Pfmdr-1* and *PfK-13* genes were amplified using a nested PCR protocol followed by Sanger sequencing.

A total of 64 *P*. *falciparum* isolates were sequenced for *Pfmdr-1* gene (NCBI Gene Bank Accession: MG641959.1 - MG642022.1). Majority of the isolates were wild type (n = 55, 85.9%); however, eight isolates (12.5%) showed a single nucleotide polymorphism (SNP) at N86Y and one (1.6%) at E130K.

With regard to *Pfcr*t gene, a total of 46 isolates (NCBI Gene Bank Accession: MH823820.1 - MH823865.1) were successfully sequenced and analysed. Of these, 87% (n = 40) of isolates carried mutant genotypes and only 13% had wild type allele. Among the mutant genotypes, all the isolates (100%, n = 40) showed triple mutations at codons M74I, N75E and K76T. Two haplotypes were identified; CVIET had 40 sequences and CVMNK had 6 sequences. Haplotype diversity was found to be 0.232 (Variance: 0.00544, SD: 0.074).

A total of sixty-two isolates were amplified for *PfK-13* by nested PCR and 48 isolates were successfully sequenced and analysed (NCBI Gene Bank accession MG366541.1-MG366588.1). No polymorphism in *K-13 propeller* gene was found that confer resistance to artemisinin-combination therapy (ACT). At the nucleotide level as well, no mutations were observed.

**Figure 1:**
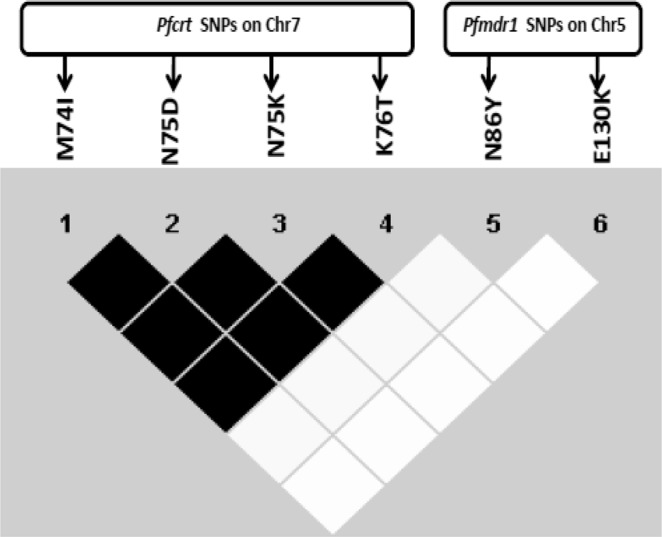
Linkage disequilibrium (LD) plot between the SNPs of *Pfcrt* and *Pfmdr1* gene in *P*. *falciparum* isolates collected from Tripura. The strength of LD between the SNPs was determined by the association of statistical significance by calculating the r^2^ values and represented with the extent of darkness of the boxes (black colour depicts strong LD and white colour depicts weak or no LD).

Linkage disequilibrium (LD) was calculated by estimating r^2^ values between all possible pairs (15 pairs) of SNPs present in *Pfcrt* and *Pfmdr-1* genes to study the presence of any intergenic or intragenic association. It was observed from the LD analysis that there is a significant intragenic association among all the 4 SNPs of *Pfcrt* locus whereas no intergenic association was observed between *Pfcrt* and *Pfmdr-1* genes from the analysis (Fig. 1).

### Genetic diversity of *P*. *falciparum* isolates

Allelic frequency, multiplicity of infection (MOI) and expected heterozygosity (H_E_) were examined in the three genetic diversity genes of *P*. *falciparum*, *viz: Pfmsp-1*, *Pfmsp-2* and *Pfglurp* using PCR.

A total of 52 clinical isolates were successfully amplified for the *Pfmsp-1*, *Pfmsp-2* and *Pfglurp* genes. Polyclonal infections were observed in 53.85% (n = 28) isolates and were significantly more common in the adult population (71.43% vs. 41.94%, *p* = 0.0494). The overall MOI for *Pfmsp-1*, *Pfmsp-2* and *Pfglurp* were 1.56, 1.31 and 1.06, respectively (Table 1).

**Table 1:** Multiplicity of infection (MOI), Expected Heterozygosity (H_E_) and frequency of allelic variants of *Pfmsp-1*, *Pfmsp-2* and *Pfglurp* genes.

Genes	Allele frequency (n = 52)	Fragment size range (bp)	Number of genotypes	Monoclonal infection (%) n = 52	Polyclonal infection (%) n = 52	chi-square *p-* value (Monoclonal vs. polyclonal infections)	MOI (n = 52)	H_E_
***Pfmsp-1***				30 (57.7%)	22 (42.3%)	0.000086	1.56	0.89
K1	37	150–290	8					
MAD20	28	150–230	4					
RO33	16	160	1					
***Pfmsp-2***				36 (69.2%)	16 (30.8%)		1.31	0.81
FC27	32	310–420	4					
3D7/IC	36	490–610	5					
***Pfglurp***	55	550–1010	9	49 (94.2%)	3 (5.8%)		1.06	0.85

The distribution of the allelic variants in children (aged ≤15 years) and adults is summarized in Table 2. MOI for *Pfmsp-1*and *Pfmsp-2* genes was observed to be higher among adults; however, for *Pfglurp*, it was higher in children (1.1 vs. 1.0).

**Table 2:** Difference in Multiplicity of infection (MOI) between adults and children.

Genes		Children ≤ 15 yrs.		Adults		*p-*value
		Allele frequency, n = 31	MOI	Allele frequency, n = 21	MOI	
*Pfmsp-1*	K1	21	1.48	16	1.67	0.5512
	MAD20	17		11		1.0
	RO33	8		8		0.3753
*Pfmsp-2*	FC27	18	1.26	14	1.38	0.5746
	3D7/IC	21		15		1.0
*Pfglurp*		34	1.1	21	1.0	0.0053

**Figure 2:**
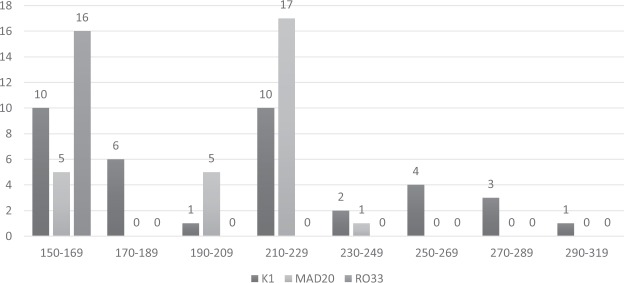
Distribution of *Pfmsp-1* (K1, MAD20 and RO33) allelic fragments.

In the *Pfmsp-1* locus, frequency of K1 allelic family was observed to be the highest among the isolates (n = 37, 71.2%) with 8 different allele fragments (range: 150–290 bp) of which the 210 bp allele was predominant (Fig. 2). While in the MAD20 and RO33 family, 53.8% (n = 28) and 30.8% (n = 16) isolates showed 4 and 1 allelic variants respectively. In addition, the 220 bp and 160 bp fragments were found to be the most predominant in MAD20 and RO33 family, respectively. The expected heterozygosity (H_E_) was found to be 0.89.

**Figure 3:**
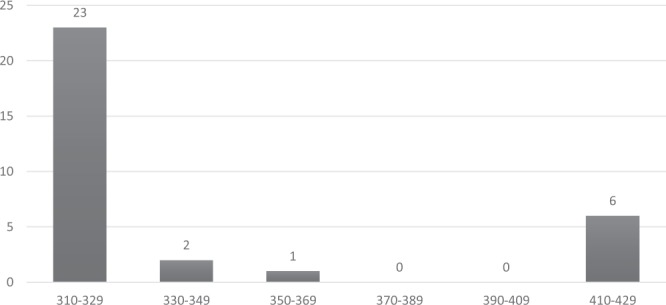
Distribution of *Pfmsp-2* (FC) allelic fragments.

**Figure 4:**
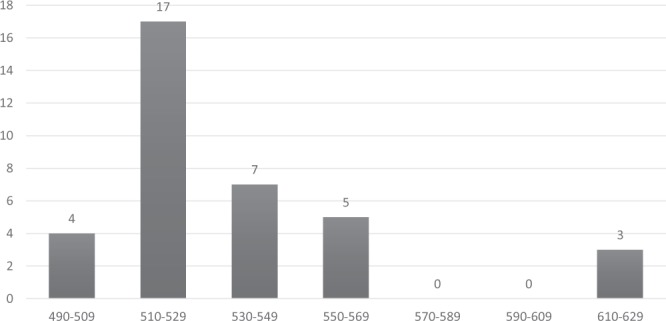
Distribution of *Pfmsp-2* (3D7) allelic fragments.

Among the *Pfmsp-2* allelic family, FC27 was detected in 61.5% (n = 32) isolates with allelic fragments ranging in size from 310–420 bp and the predominant allelic fragment was found to be 310 bp in size (Fig. 3). The 3D7/IC allelic family was detected in 69.2% (n = 36) isolates and the 520 bp fragment was found in 36.1% (n = 13) isolates (Fig. 4). The expected heterozygosity (H_E_) was found to be 0.81.

**Figure 5:**
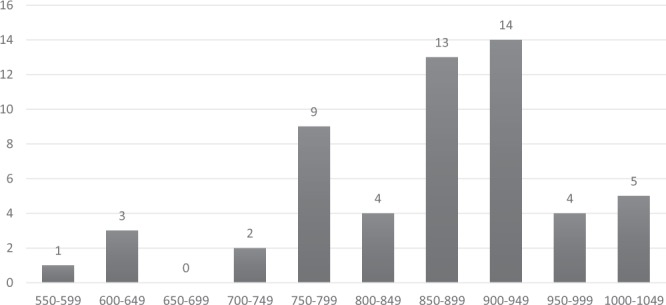
Distribution of *Pfglurp* allelic fragments.

A total of nine different allelic fragments (550–1010 bp) were observed in the RII region of *Pfglurp* (Fig. 5). The 850 bp fragment was the most commonly detected allele (n = 11, 21.15%). The expected heterozygosity (H_E_) was found to be 0.85.

**Figure 6:**
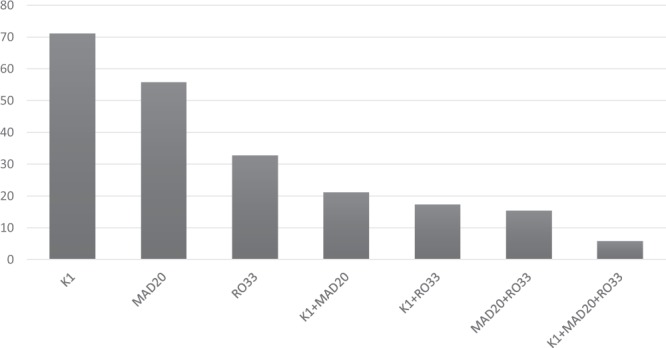
Frequency of *Pfmsp-1* allelic fragment combinations (in percentages).

The frequencies of the allelic variants of the three genetic loci are shown in Table 1. MOI was found to be highest for *Pfmsp-1*, where the highest number (42.3%) of polyclonal infections was also detected. In the polyclonal infections, the following allelic combinations were observed in *Pfmsp-1* gene: K1/MAD20 in 21.2% (n = 11) isolates, K1/RO33 in 17.3% (n = 9) isolates, MAD20/RO33 in 15.4% (n = 8) isolates and K1/MAD20/RO33 (triple allele) in 5.8% (n = 3) clinical isolates (Fig. 6). For *Pfmsp-2* gene, the FC27/3D7 alleles in combination were present in 16 isolates. In the *Pfglurp* locus, 5.8% (n = 3) isolates were found to be polyclonal, all of which occurred in children ≤15 years of age.

### Correlation between Polyclonality of infection and mutation in anti-malarial drug resistance genes

Out of the 71 *P*. *falciparum* positive cases, 35 samples with complete data for both the parasite genetic diversity markers (*Pfmsp-1*, *Pfmsp-2 and Pfglurp*) and drug resistance gene markers (*Pfcrt* and *Pfmdr-1*) were analysed to explore any association between polyclonality and anti-malarial drug resistance. It was observed that 95.5% of the polyclonal infections harboured a *Pfcrt* mutation whereas for monoclonal infections, the rate was 76.9% (p = 0.26474). Similarly, it was found that 13.6% of the polyclonal infections harboured a mutation in the *Pfmdr-1 gene* while only 7.7% of the monoclonal infections had the same; the differences were however, not statistically significant (Table 3).

**Table 3:** Correlation between Polyclonal infections and mutation in drug resistance genes (*Pfcrt* and *Pfmdr-1*).

	*Pfcrt* Mutation		*p*-value (2 Tailed P Test)	*Pfmdr-1* Mutation		*p*-value (2 Tailed P Test)
	Yes	No		Yes	No	
Polyclonal	21	1	0.26474	3	19	1.0
Monoclonal	10	3		1	12	

## Discussion

The current study was carried out to characterize the major molecular markers governing anti-malarial drug resistance and genetic diversity in *P*. *falciparum* isolates obtained from a highly malaria endemic area of Tripura in Northeast India. The status of drug resistant parasite strains in a community and the diversity of the parasite population are important determinants in understanding the molecular epidemiology of malaria, especially in high endemic areas.

The *Pfcrt* protein is localised on the digestive vacuole of the malaria parasite and functions as an anion channel to mediate the efflux of CQ outside the vacuole^11^. Mutation in the *Pfcrt* domain, specifically the replacement of lysine 76 with threonine is the most widely described marker for CQ resistance^12^. This mutation leads to an increase in the lipophilicity and negativity of the *Pfcrt* protein and specifically favours the efflux of positively charged CQ fractions outside the digestive vacuole. Lysine, having a net positive charge and possessing a bulkier side chain as compared to threonine, inhibits the efflux of CQ fractions from the digestive vacuole^11^. The *Pfmdr-1* protein is also a transporter on the digestive vacuole membrane of the malaria parasite and it normally mediates the transfer of drugs such as CQ from the cytosol into the digestive vacuole. Single nucleotide polymorphisms in the *Pfmdr-1* gene such as the N86Y mutation lead to changes in the physicochemical properties of the transporter thereby altering its ability to bind and transfer the target drugs^11^.

Originating from the Thai-Cambodia border in the 1950s to its appearance in Assam, Northeast India in 1973, CQ resistance has spread extensively in other parts of India^12,13^. In Assam, during 2006–2007, prevalence of the mutant *Pfcrt* genotype (K76T) and *Pfmdr-1* genotype (N86Y) was 99% and 68%, respectively^13^. The widespread resistance warranted a change in the anti-malarial drug policy in India with Artemisinin based combination therapy (ACT) replacing CQ for uncomplicated falciparum malaria in 2008^14^. In the present study, prevalence of *Pfcrt* mutation was observed to be high (87%) as compared to previous studies from India^15,16^. In addition, majority of our isolates harboured the triple mutant genotype (C_72_V_73_I_74_E_75_T_76_) and only 13% isolates carried wild type genotype, which is in agreement with previous Indian studies after CQ withdrawal^15,16^. In this scenario, the efficacy of implementation of ACT needs to be explored in the region; the possibility that CQ might still be used unofficially for uncomplicated *P*. *falciparum* malaria is high and needs to be evaluated. With the current emphasis on the return of CQ sensitive parasites in malaria endemic regions after the withdrawal of drug pressure for sufficient time, the present finding of a high rate of *Pfcrt* mutations in Tripura represents a challenge for the future control of malaria in the region where resistance to ACT might become a reality. The changing trend of CQ efficacy and *Pfcrt* mutations after drug withdrawal is interesting. The Comoro islands reported a dramatic reduction in the *Pfcrt* K76T mutation after CQ withdrawal^17^. Similar trends were also observed in Ghana, Kenya, Malawi and other African countries^18–20^. This is encouraging and reinforces the possibility of reintroduction of CQ in the future for malaria control. However, contrasting findings have also been reported from Gabon and Benin where the frequency of *Pfcrt* K76T mutant was still high after four to seven years of CQ withdrawal^21,22^. Therefore, a constant monitoring of CQ resistance along with compliance and adherence of anti-malarial drug administration is highly required to assess the dynamics of CQ resistance in an area.

The prevalence of N86Y mutation in the *Pfmdr-1* gene was 12.5%, which is lower than other studies from Assam, Chhattisgarh and Puducherry, but comparable to figures reported from Orissa^13,15,16^. Although the *Pfcrt* and *Pfmdr-1* gene mutations have been linked to CQ resistance, their role is still unclear and many studies have failed to find any association^16,23,24^.

In this study, LD analysis revealed significant intragenic association between SNPs detected in the *Pfcrt* locus, while no intergenic association with *Pfmdr-1* was noted. This is in agreement with previous studies from other parts of India^16,25^.

With the current use of ACTs for *P*. *falciparum* malaria throughout the world, monitoring anti-malarial drug resistance and treatment efficacy is important for malaria control. In recent years, Kelch 13 propeller domain based molecular surveillance technique has become an important tool and is widely used^7^. In the current study, no polymorphisms were noted, concurrent with previous reports from other parts of India^15,26^. This however, needs to be evaluated further as non-synonymous mutations in the *K13* propeller domain were reported from this area earlier but were not associated with ACT treatment failure^27,28^. Recently, Chakrabarti *et al*., demonstrated reduced artemisinin sensitivity in the north-eastern isolates of *P*. *falciparum* by using ring stage survival assay demanding a future investigation on the potential change in the ACT effectiveness in *P*. *falciparum* parasites from NE India^29^.

In the milieu of the prevailing polymorphisms detected in the anti-malarial drug resistance genes, the current study also analysed the diversity patterns in potential vaccine candidate genes of *P*. *falciparum* parasites (*Pfmsp-1*, *Pfmsp-2* and *Pfglurp*) circulating in the region. The genetic polymorphism analysis presents an interesting prospect for the future adoption of a successful vaccine candidate in this part of the country^30^. In addition, earlier studies have shown that highly complex malaria infections, as demonstrated by high allelic diversities and polyclonal infections have a propensity to select drug resistant parasites and cause more virulent infections^10,31,32^. In the present study, *Pfmsp-1* locus exhibited 13 different genotypes and K1 allelic family was the most frequently observed followed by MAD20 and RO33. This finding is in contrast with previous studies from neighbouring Arunachal Pradesh and Chandigarh, North India where RO33 allelic frequency was highest followed by MAD20 and K1^33,34^. However, MAD20 was found to be more frequent than K1 and RO33 in Central India and Bangladesh^35,36^.

Hitherto, a wide range of diversity patterns in the block-2 region of *Pfmsp-1* and their correlation with disease severity and endemicity patterns have been reported worldwide^37,38^. Ranjit *et al*. in Orissa, India demonstrated an association of the 200 bp allelic fragment of MAD20 and 550 bp allele of 3D7 with severe malaria cases^39^. The block-2 region of *Pfmsp-1* contains degenerate tripeptides and repeat sequences which have been shown to participate in recognition of erythrocyte surface and incorporation into the red cell cytoskeleton in malaria pathogenesis^40^. Specific allelic forms of *Pfmsp-1* in isolation or in combination with other markers might favour the expedited entry of the malaria parasite into the red cells thereby favouring rapid multiplication, high parasitaemia and more severe disease^39,41^. However, this association could not be ascertained in our study as patient follow-up was not a part of the study protocol.

In the present study, the *Pfmsp-2* family with 9 different allelic forms was found to be less polymorphic as compared to the *Pfmsp-1* allelic family. In addition, within the *Pfmsp-2* family, the 3D7 component was more abundant as compared to FC27 with both the allelic variants occurring together in 30.8% isolates, which corroborates with earlier reports from Myanmar and Cameroon^42,43^. The presence of 13 and 9 different allelic forms for *Pfmsp-1* and *Pfmsp-2* respectively is concurrent with previous studies from mesoendemic to hypoendemic Asian countries like Thailand and Iran^44,45^. The R2 region of *Pfglurp* was observed in all our isolates and showed considerable polymorphism with 9 allelic variants with frequencies ranging from 1.8% to 25.5%; similar findings were obtained from Arunachal Pradesh and Assam, Northeast India^33,46^. Additionally, the greater number of allelic variants with low allele frequencies of *Pfglurp* encountered in the present study is suggestive of high endemicity in the area. Regions with low malaria endemicity like Central and South American countries were found to have two to four *Pfglurp* (R2) alleles^47,48^. However, most of the studies from highly endemic areas in Asia and Africa have reported eight to twenty *Pfglurp* (R2) alleles^39,44,46,49^.

Multiplicity of infection has been shown to be invariably related to transmission intensity and parasite prevalence, but results are still inconclusive^50^. In the current study, MOI for each of the three selected polymorphic antigenic genes ranged from 1.06 to 1.56, the highest being noted for *Pfmsp-1* and lowest for *Pfglurp*. These results are in agreement with earlier studies from Chhattisgarh, central India and Udalguri district of Assam where malaria is endemic^35,46^. In recent years, two major changes were recommended in anti-malarial drug policy in Northeast region; the first was the introduction of ACT in 2010 and the second was the replacement of Artemether and sulphadoxine-pyrimethamine combination (A + SP) with Artemether-Lumefantrine (A + L) in 2013. The MOI over this period has remained largely static; the slight reduction might be attributable to the change in drug policy or other vector control measures^46^. African studies conducted in different transmission settings have reported the lowest MOI rates from areas of low transmission and highest MOI figures from regions where malaria transmission was perennial^51^. In the present study, we found a higher MOI among adult population with regard to the *Pfmsp-1* and *Pfmsp-2* antigenic markers, which points to greater exposure to infection with increasing age. To date, studies have reported both similar and conflicting observations and some have found no association of MOI with age^52–54^.

Heterozygosity values observed in our study for the individual antigenic markers were slightly higher than those reported earlier from Southeast Asia/Pacific and South American countries, but concurrent with those seen in African locations^51,54^. The H_E_ values of *Pfglurp* were concurrent with previous reports from Assam^46^. The high H_E_ values suggest a high transmission rate and a comparatively large parasite population circulating in the region. In this scenario, the possibility of genetic recombination of the parasite strains in mosquito vectors is also expected to be considerable^55^. The involvement of the indigenous tribes in Jhum, a shifting form of cultivation also results in a high rate of man-mosquito contact in this region thereby facilitating this kind of interaction^56^.

The current study documented a high rate of polyclonal infections with comparatively higher rates of mutation in *Pfcrt* and *Pfmdr-1* genes as compared to that observed in monoclonal infections. The presence of infections with multiple alleles is indicative of considerable genetic diversity in the parasite population which in turn can lead to the emergence and proliferation of drug resistant clones^31,57^. Similarly, MOI and polyclonality of *P*. *falciparum* can also be considered as an indicator of malaria control efforts in an area, as demonstrated by Hetzel *et al*., where a higher MOI and polyclonality was found to be associated with an area of no intervention^58^. A positive association was observed between the rate of polyclonal infections and annual parasite incidence in Indonesia indicating that polyclonality of *P*. *falciparum* over an area might provide information on local transmission intensity^59^. Moreover, in most polyclonal infections, there are invariably large populations of drug sensitive parasites which mask the detection of small populations of drug resistant strains (minority variants) by standard PCR; accurate detection requires more sensitive methods as demonstrated in Malawi^60,61^.

In the context of high parasite diversity and circulating population of drug resistant strains, whether the genetic structure of the patients themselves residing in this area has a role to play is another interesting aspect. Historically, the indigenous tribes of Tripura belong to the Tibeto – Burman language family which is more or less a homogenous group with high rates of endogamy^62,63^. Previous studies carried out in African ethnic groups have demonstrated that both immune response and susceptibility to malaria infection might vary depending on the genetic background of the study population. Even drug resistance patterns can vary with genetic polymorphism patterns in host enzymes^64^. Similar information from malaria endemic areas of India would provide valuable leads for control measures.

Tripura faced an epidemic of malaria in 2014 with an increase of almost seven-fold *P*. *falciparum* malaria cases over 2013^65^. It has been observed that malaria in Tripura was in a state of decline till 2014. However, the epidemic has reset the baseline case load of malaria in the state at a higher level than before. A clonal population structure with identical genotype of *P*. *falciparum* is expected to circulate during the epidemic period^66^. However, what drives the changes in the clonal structure and diversity of parasites in the post epidemic period is still not clear.

The limitations of the present study include (i) inability to correlate polymorphism patterns observed in anti-malarial drug resistance genes with treatment response or clinical outcome and (ii) a relatively small sample size. Nevertheless, the strength of the current study lies in the adoption of robust protocols for characterization of the major drug resistance and genetic diversity genes of *P*. *falciparum*. Although the number of malaria positive cases included in the characterization of the genetic markers was not very high, there was no obvious sampling bias and sufficient care was taken to cover two of the most highly malaria endemic districts of Tripura. Cases were included following proper ethical guidelines and treatment was also provided to the patients as per national guidelines.

In conclusion, the present study showed a high level of polymorphism in the genes governing CQ resistance in the region even after a significant period of drug withdrawal as compared to other malaria endemic areas of India and Africa. No polymorphisms were found in the *PfK13* domain, which, however, needs to be evaluated further in the milieu of extensive genetic diversity observed in the parasite population. The polymorphic regions of *Pfmsp-1*, *Pfmsp-2* and *Pfglurp* genes exhibited high allelic diversity suggestive of high malaria endemicity and high heterozygosity in the study area. All these factors are conducive to the gradual selection and proliferation of drug resistant parasites and may make malaria more difficult to control in this region in the days to come.

## Materials and Methods

### Study areas and design

This study was carried out in the malaria endemic areas of North Tripura and Dhalai districts of Tripura state (located between Lat. 23.9408°N and Long. 91.9882°E), Northeast India in May 2015. The population composition in these areas is primarily tribal, with Tripuri, Reang and Chakma tribes predominating. Symptomatic patients were recruited as per the following inclusion criterion: body temperature ≥37.5 °C, age >1 year, history of fever within one week, no recent history of consumption of anti-malarial drugs and absence of severe malnutrition or signs of severe malaria. Symptomatic patients were screened for *Plasmodium* parasites using rapid diagnostic test (RDT) and microscopic examination of thick and thin blood smears stained with Jaswant Singh and Bhattacharjee (JSB) stain. Two ml of whole blood was collected from malaria positive patients after informed written consent. All malaria positive patients were given treatment with anti-malarial drugs as per the National Vector borne Disease Control Programme (NVBDCP) guidelines for NE India^67^.

### Ethical approval

The current study was approved by the Institutional Ethics Committee (Human) of ICMR - RMRC N. E. Region, Dibrugarh, Assam (Ref: RMRC/Dib./IEC Human/2012/667) and all study protocols and procedures were carried out according to guidelines of Indian Council of Medical Research (ICMR). Written informed consent was obtained from the study participants (parents of participants in case of minors) prior to collection of blood samples.

### DNA extraction and nested PCR for malaria parasite species identification

Parasite genomic DNA was extracted from whole blood samples using the QIAamp DNA blood mini kit as per manufacturer’s instructions (Qiagen, CA, USA). The extracted DNA was stored at −20 °C for further analysis. Conventional nested PCR was performed using pan-*plasmodium* and species-specific primers for molecular identification of the malarial parasites as described previously^68^.

### Genotyping of drug resistance and vaccine candidate genes

All samples which tested positive for *P*. *falciparum* were subsequently subjected to further molecular characterisation. The drug resistance markers; *PfK13*, *Pfmdr1* and *Pfcrt* genes were amplified and sequenced as described previously^7,23,69^. The PCR products were purified using a column based purification protocol (High Pure PCR Product Purification Kit, Roche) and sequenced using the Sanger’s technique^70^. The sequences were edited using BioEdit ver 7.2.5 software and aligned using the ClustalW multiple alignment tool built into the software. Edited sequences were submitted to the NCBI GeneBank. The DNA sequences were translated using the ExPASy portal and the amino acid sequences generated were tallied with NCBI database reference sequences using ClustalW^71^.

The genetic polymorphism in *Pfmsp-1*, *Pfmsp-2* and *Pfglurp* genes was examined using allele specific nested PCR as previously described without the aid of nucleotide sequencing^72^. Specifically, the K1, MAD20 and RO33 allelic families of *Pfmsp-1* block 2, FC27 and 3D7/IC families of *Pfmsp-2* block 3 and Region 2 (RII) of *Pfglurp* were amplified using primers specific for the individual allelic variants. Amplified PCR products were subjected to agarose gel electrophoresis and stained with EtBr followed by visualisation in a BioRad XR gel documentation system. Laboratory adapted strains, Dd2 and 3D7, were used as controls.

### Multiplicity of infection and heterozygosity

The grouping of the different fragments based on amplified DNA product size was done as described previously for determination of multiplicity of infection (MOI); for *msp1* and *msp2*, alleles having a size difference within 20 bp were considered the same, while for *Pfglurp*; a larger interval of 50 bp was considered^73^. A monoclonal infection was identified by the presence of a single PCR band for each locus and a polyclonal infection was defined as the presence of multiple PCR fragments for any of the three loci. Multiplicity of infection was defined as the average number of different parasite genotypes (denoted by the maximum number of bands detected for either loci) infecting a single host simultaneously^54^. Expected heterozygosity (H_E_) which denotes the possibility of being infected simultaneously by two parasites with different alleles at a given locus was estimated by using the following formula: H_E_ = [n/(n − 1)] [(1 − Σpi^2^)], where ‘n’ is the total number of samples tested and ‘pi’ is the frequency of the allele (%) at the given locus^54^.

### Statistical analysis

Statistical analyses were performed using EpiInfo ver. 7.2.2.6. Fisher’s exact test and 2 tailed test were used for determining the significance of monoclonal versus polyclonal infections and relationship of the diversity genes with age. Statistical significance was considered at a *p* value < 0.05. MOI was calculated independently for each gene by dividing the total number of alleles obtained for a given gene (*Pfmsp-1*, *Pfmsp-2*, *Pfglurp*) by the number of isolates positive for that gene by PCR^54^. To determine the association between the SNPs detected in the drug resistance genes, both intergenic and intragenic linkage disequilibrium (LD) analysis was done using the program Haploview^74^.

## Data Availability

The article text includes all data generated in the course of the study. Submitted sequence IDs have been mentioned in the text and are available in the NCBI database and also with the corresponding author.
